# Are C-reactive protein and procalcitonin safe and useful for antimicrobial stewardship purposes in patients with COVID-19? A scoping review

**DOI:** 10.1017/ash.2024.372

**Published:** 2024-09-12

**Authors:** Anita Williams, Ernestina Repetto, Ishmael Lebbie, Mohamad Khalife, Tomas Oestergaard Jensen

**Affiliations:** 1 Wesfarmers Centre for Vaccines and Infectious Diseases, Telethon Kids Institute, Perth, Australia; 2 Middle East Medical Unit, Médecins Sans Frontières, Beirut, Lebanon; 3 Luxembourg Operational Research (LuxOR) Unit, Médecins Sans Frontières, Luxembourg City, Luxembourg; 4 Infectious Diseases Department, Université Libre de Bruxelles (ULB), CHU Saint-Pierre, Brussels, Belgium; 5 Kenema Project, Médecins Sans Frontières, Operational Centre Brussels, Kenema City, Sierra Leone; 6 Medical Department, Médecins Sans Frontières, Operational Center Paris, Paris, France; 7 Center of Excellence for Health, Immunity, and Infections, Rigshospitalet, University of Copenhagen, Copenhagen, Denmark

## Abstract

**Objective:**

The primary objectives of this study were to assess the usefulness of C-reactive protein (CRP) and procalcitonin (PCT) in the diagnosis of bacterial co-infections in coronavirus disease 2019 (COVID-19) and if their incorporation in antimicrobial stewardship (AMS) programs is safe and useful, stratified by severity of disease as level of care, intensive care unit (ICU) or non-ICU. Our secondary objectives were to identify cut-off values for antibiotic decision-making and identify reported results from low- and middle-income countries (LMICs).

**Design:**

A scoping review of published literature, adhering to the PRISMA statement for Systematic Reviews and Meta-analyses Extension for Scoping Reviews guidelines. The last search was performed in January 2024.

**Results:**

Fifty-nine studies were included in this scoping review: 20 studies reporting predictive values and/or sensitivity/specificity results for PCT, 8 reporting clear objectives on AMS, and 3 studies from LMICs.

**Conclusion:**

In the context of non-ICU hospitalized COVID-19 patients in high-income countries, a PCT value below 0.25 mg/L can be a useful tool to rule out bacterial co-infection. The wide range of reported negative predictive values suggests that PCT should be interpreted in the context of other clinical findings. Our results do not support the use of CRP in the same manner as PCT. There is a clear need for more studies in LMICs.

## Introduction

The capacity to diagnose bacterial co-infections in patients with coronavirus disease 2019 (COVID-19) is limited by laboratory capacity, especially in low- and middle-income countries (LMICs) where access to microbiology is sparse. The large caseload of COVID-19 has resulted in global concerns about increasing empiric antibiotic usage and potential setbacks for antimicrobial stewardship (AMS) programs. This is particularly acute in low-resource settings without access to extensive microbiological testing. There is a real risk that new waves of COVID-19 may drive an increase in antimicrobial resistance, and there is a need for tools to guide optimal antimicrobial prescribing and stewardship.^1^

Host inflammatory biomarkers, such as procalcitonin (PCT) and C-reactive protein (CRP), have been proposed as possible indicators for distinguishing between viral and bacterial infections.^2–4^ Serum PCT levels in healthy individuals are usually <0.05 ng/mL; in most bacterial infections, the concentration increases in proportion with the severity of the illness.^5,6^ In most viral infections, increased interferon gamma production inhibits PCT synthesis, leading to relative bacterial specificity of PCT.^7^ However, there are clinical situations that may lower this specificity, including patients on medications that stimulate cytokine release, chronic kidney disease, major surgery, or severe trauma.^5,8^ Normal CRP levels in most healthy adults are usually <10.0 mg/L. There are both acute and chronic conditions and infectious and noninfectious etiologies for an elevated CRP level. However, CRP levels rise and fall rapidly with the introduction and removal of inflammatory stimuli. These and other factors mandate the interpretation of biomarkers within the context of other laboratory and clinical findings.^9,10^

Prior to the pandemic, the Food and Drug Administration in the United States had advised on cut-off values for PCT-guided antibiotic use in lower respiratory tract infections, as well as international consensus on the use of PCT in combination with clinical patient assessment for AMS algorithms.^10–12^ During the COVID-19 pandemic, several guidelines were published suggesting the use of biomarkers for AMS purposes—the United Kingdom (UK) first published the NICE rapid guidance NG173 on May 1, 2020.^13^ However, whether biomarkers could be used as an indicator of secondary bacterial infection and need for antibiotics in severe acute respiratory coronavirus virus 2 (SARS-CoV-2) positive patients is a more specific question that needs to be answered, especially for LMICs.

Therefore, the primary objectives of this study were to assess the usefulness of CRP and PCT in the diagnosis of bacterial co-infections in COVID-19 and if their incorporation in AMS programs is safe and useful, stratified by severity of disease as level of care, intensive care unit (ICU) or non-ICU. Our secondary objectives were to identify cut-off values for antibiotic decision-making and identify reported results from LMICs.

## Methods

This was a scoping review of published literature, adhering to the PRISMA statement for Systematic Reviews and Meta-analyses Extension for Scoping Reviews guidelines.^14^ Studies published from January 2020 onward in all languages were considered eligible for screening. The following key terms were included: “COVID and/or SARS-CoV-2,” “antibiotic stewardship” *or* “antimicrobial stewardship,” “bacterial co-infection,” and biomarkers *or* procalcitonin/PCT *or* C-reactive protein/CRP. The last search was performed in January 2024. The PubMed, Scopus, EMBASE, and Web of Science databases were used to identify relevant literature. Search histories were uploaded in Covidence, a web-based collaboration software platform that streamlines the production of systematic and other literature reviews.^15^

Initial abstract review and full text screening was performed in duplicate by AW and ER, with conflicts resolved by TOJ. Extraction of data was performed in duplicate by any 2 of the authors (AW, ER, MK, TOJ), with a third performing a consensus check. The reference lists of key systematic review articles were also manually searched for studies not identified through electronic searches.^3,4,8^

Studies that did not address the diagnosis of bacterial co-infections in COVID-19 specifically, or a stewardship program, were excluded. Additionally, studies that only explored the use of biomarkers as predictors of severity, clinical outcomes, or length of stay were excluded.

## Results

**Figure 1.**
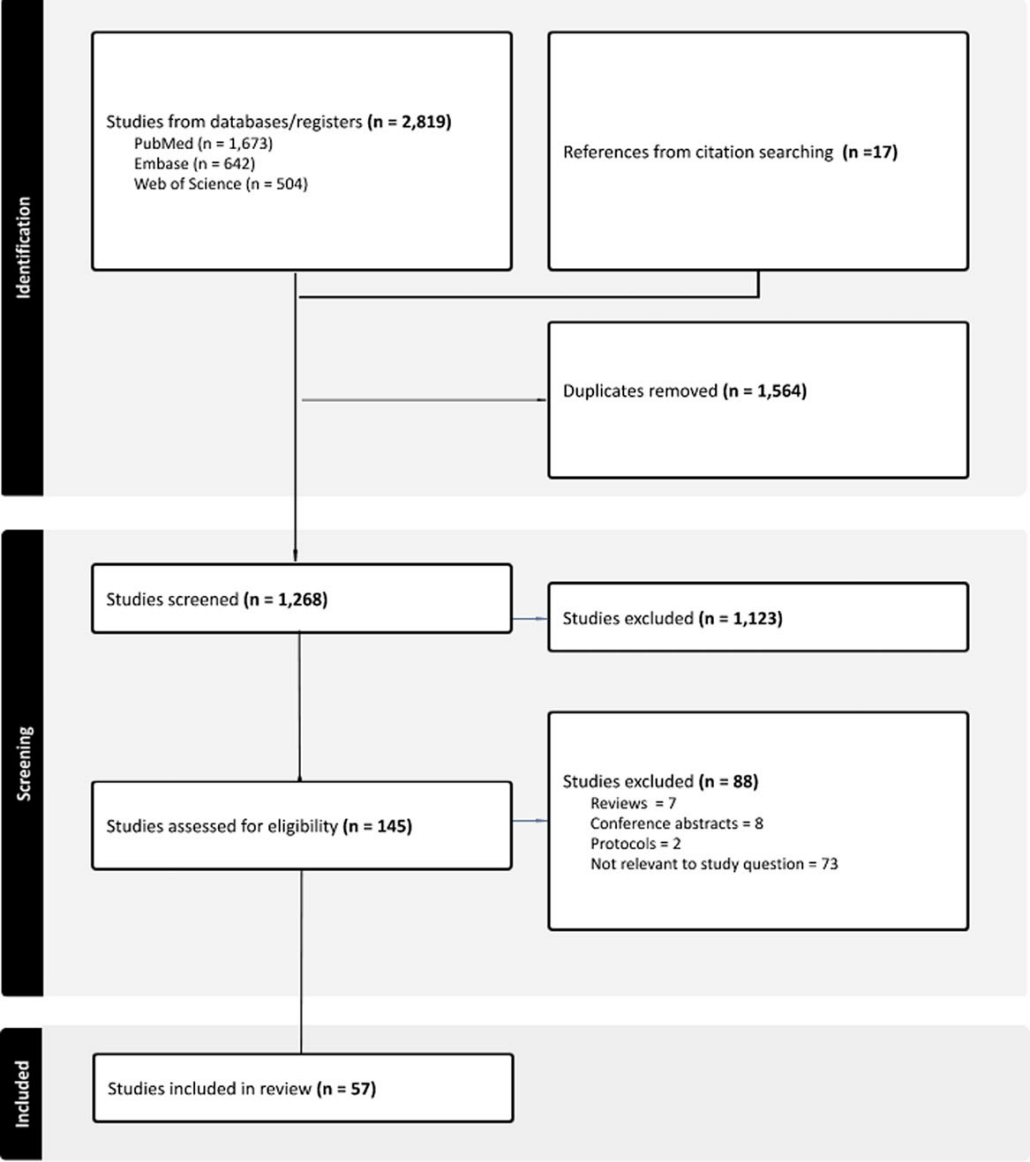
Flow diagram for scoping review process.

Figure 1 displays the flow diagram describing the article selection process. Initially, 2,819 references were imported for screening with an additional 17 references from citation searching of key review articles. Once titles and abstracts were screened, 145 articles were evaluated for inclusion, leading to 59 studies being included in the review.

Of the 59 studies selected, 48 were conducted in high-income countries (HICs) with 14 studies conducted in the United States and 11 conducted in the UK. Seven studies were conducted in upper middle-income countries (UMICs); 5 studies were from China, and 1 was a multicenter study conducted concurrently in several HICs and UMICs. Three studies were conducted in LMICs: 1 each from India, Nepal, and Pakistan (Table 1).

**Table 1.** Characteristics of studies included in this review

First author, (year published)	Location/s	World Bank category^16^	Start	Study type	Biomarker used	Sample size	ICU/non-ICU	Secondary infection type	% Sec. bacterial infection
Anderson (2023)^17^	USA	HIC	Oct 2020 and Jan 2021	Single-center before-and-after observational	PCT	298	–	Respiratory	8.8 (pre) 9.7 (post)
Antuori (2023)^18^	Spain	HIC	March 2020–April 2021	Retrospective observational	PCT	1,157	Both	Respiratory	6.2 (non-ICU) 8.9 (ICU)
Atallah (2022)^19^	USA	HIC	17/03/2020–30/04/2020	Retrospective case control	PCT	324	Both	Both	39.5
Azijli (2022)^20^	The Netherlands	HIC	Jan–April 2019 and Jan–April 2020	Prospective observational cohort	PCT	546	Non-ICU	Non-respiratory	3.8
Basnet (2022)^21^	Nepal	LMIC	25/06/2021–24/12/2021	Retrospective cross-sectional	PCT	49	–	Non-respiratory	6.1
Bhatt (2021)^22^	USA	HIC	01/03/2020–07/05/2020	Retrospective multicenter case control	CRP	375	Both	Non-respiratory	7.1
Calderon (2021)^9^	UK	HIC	12/03/2020–01/07/2020	Retrospective single- site cohort	PCT	259	Non-ICU	–	7.7
Campani (2023)^23^	Italy	HIC	Feb 2020–May 2022	Retrospective cohort	CRP, PCT	279	ICU	Both	60.6
Carbonell (2022)^24^	Colombia, Chile, Ecuador, Mexico, Argentina, Uruguay, Brazil, Spain, Ireland, Andorra	HIC; UMIC	March 2020–Jan 2021	Retrospective cohort	PCT	4,365	ICU	Respiratory	7.6
Ceccarelli (2023)^25^	Italy	HIC	March 2020–Feb 2021	Retrospective observational	PCT	184	ICU	Both	36.4
Cheng (2020)^26^	China	UMIC	10/01/2020–09/03/2020	Prospective observational	PCT	212	–	Both	14.6
Cheng (2020)^27^	Hong Kong	HIC	08/01/2020–01/05/2020	Retrospective cohort	CRP	147	Both	Both	8.2
Cidade (2023)^28^	Portugal	HIC	01/01/2020–31/03/2021	Prospective cohort	CRP, PCT	118	ICU	Both	29.7
Conlon (2022)^29^	USA	HIC	01/03/2020–31/10/2021	Retrospective observational	PCT	793	–	Respiratory	14.7
Côrtes (2021)^30^	Brazil	UMIC	April 2020–June 2020	Prospective cohort	CRP, PCT	73	ICU	Respiratory	38.4 (VAP)
Cowman (2022)^31^	USA	HIC	March 2020–April 2020	Retrospective observational	CRP, PCT	819	Both	Both	8.9
Dolci (2021)^32^	Italy	HIC	Feb 2020–March 2020	Retrospective observational	CRP, PCT	83	Both	Both	39.8
Fabre (2021)^33^	USA	HIC	01/03/2020–30/05/2020	Retrospective descriptive	CRP, PCT	962	Both	Both	1
Fartoukh (2023)^34^	France	HIC	20/04/2020–23/11/2020	Multicenter, parallel-group, open label, randomized controlled trial	PCT	194	ICU	Both	48.4
Fratoni (2022)^35^	USA	HIC	01/11/2020–26/02/2021	Retrospective quasi-experimental	PCT	772	Non-ICU	–	8.9
Galli (2023)^36^	Spain	HIC	05/02/2020–21/12/2021	Retrospective sub-analysis from prospective cohort	CRP, PCT	4,076			CO: 3.3
Garrido (2021)^37^	Spain	HIC	March 2020–May 2020	Retrospective observational cohort	PCT	56	Both	Both	44.6
Gianella (2022)^38^	Italy	HIC	Feb–Dec 2020	Multicenter observational study	CRP, PCT	1,733	Both	Both	6.3
Harte (2023)^39^	Wales	HIC	17/11/2020–15/03/2021 and 03/03/2021–22/02/2022	Retrospective observational	CRP, PCT	238	ICU	Both	51.1
He (2021)^40^	China	UMIC	10/02/2020–28/02/2020	Retrospective multicenter observational	CRP, PCT	905	Both	–	9.5
Heer (2021)^41^	UK	HIC	Feb 2020–Sept 2020	Retrospective observational	CRP, PCT	60	ICU	Both	43.3
Heesom (2020)^42^	UK	HIC	06/04/2020–22/05/2020	Prospective single-center cohort	PCT	52	ICU	–	–
Hessels (2023)^43^	The Netherlands	HIC	Oct 2020–July 2021	Retrospective multisite cohort	PCT	759	Both	Both	CO: 0.9%
Houghton (2021)^44^	UK	HIC	05/03/2020–26/04/2020	Retrospective observational	CRP, PCT	224	Both	–	3.6
Hughes (2021)^45^	UK	HIC	01/12/2020–28/02/2021	Retrospective observational	CRP, PCT	624	–	Both	CO: 3.2
Kubin (2021)^46^	USA	HIC	02/03/2020–31/05/2020	Retrospective cohort	CRP, PCT	3,028	Both	Both	CO: 6 HO: 12
Lee (2023)^47^	South Korea	HIC	Feb 2020–Dec 2021	Retrospective observational	CRP	300	Both	Both	8.3
Lingscheid (2022)^48^	Germany	HIC	March 2020–Nov 2020	Prospective observational cohort	CRP, PCT	309	Both	Both	*BC:* CO: 4.3 HO: 15.2 *RC:* CO: 32.4 HO: 29.4
Lukose (2024)^49^	India	LMIC	01/03/2021–01/08/2021	Retrospective observational	CRP, PCT	525	Both	Both	18.1
Malinverni (2022)^7^	Belgium	HIC	01/03/2020–31/10/2020	Case-control observational	PCT	359	Non-ICU	Respiratory	–
Mason (2021)^50^	UK	HIC	01/03/2020–31/05/2020	Retrospective cohort (two hospitals)	CRP	1,075	NA	Respiratory	4.2 (site 1) 5.5 (site 2)
May (2021)^51^	USA	HIC	10/03/2020–30/06/2020	Retrospective cohort	CRP, PCT	2,443	Non-ICU	Both	6.1
Ming (2021)^1^	UK	HIC	01/03/2020–06/05/2020	Retrospective descriptive	CRP, PCT	237	Both	Both	28
Moffitt (2023)^52^	USA	HIC	15/03/2020–31/12/2020	Retrospective cohort	CRP, PCT	532	ICU	Both	7.1
Moore (2022)^53^	USA	HIC	01/06/2020–31/07/2020	Retrospective cohort	PCT	173	Both	Both	15.6
Moreno-García (2022)^54^	Spain	HIC	19/02/2020–24/02/2021	Observational cohort	CRP, PCT	1,125	NA	Both	9.10
Nasir (2021)^55^	Pakistan	LMIC	Feb 2020–June 2020	Retrospective case control	CRP, PCT	100	Both	Both	N/A
Nazerian (2021)^56^	Italy	HIC	07–16 April (pre) and 18–27 April 2020 (post)	Prospective, single-center before-and-after observational	PCT	444	Non-ICU	–	1.1
Ng (2022)^57^	Singapore	HIC	22/01/2020–15/04/2020	Retrospective cohort	CRP, PCT	717	Both	Both	7.5
Peters (2020)^58^	UK	HIC	08/04/2020–27/04/2020	Quality improvement project	PCT	118	Non-ICU	–	–
Pham (2023)^59^	USA	HIC	01/11/2020–31/01/2021	Retrospective descriptive	PCT	199	Both	Both	CO: 3.0 HO: 9.5
Pink (2021)^60^	Germany	HIC	06/03/2020–30/10/2020	Retrospective single center	CRP, PCT	99	Both	Both	BC: 14% BAL 29 HAI: 32
Relph (2022)^61^	UK	HIC	06/02/2020–08/06/2020	Retrospective sub-cohort analysis from a prospective study	CRP, PCT	1,040	Both	Both	BC: 17.3 RC: 3.5
Richards (2021)^62^	UK	HIC	09/03/2020–05/06/2020	Retrospective observational	CRP, PCT	65	ICU	Both	50.8
Roy (2022)^63^	USA	HIC	01/03/2020–14/08/2020	Retrospective chart review	PCT	147	NA	Both	BC: 8.8 RC: 17
Sathitakorn (2022)^64^	Thailand	UMIC	01/04/2021–08/08/2021	Prospective cohort	PCT	120	ICU	Both	–
Tang (2021)^65^	China	UMIC	28/01/2020–15/03/2020	Retrospective study	CRP, PCT	78	Both	Respiratory	14.1
Tanzarella (2023)^66^	Italy	HIC	March 2020–June 2022	Multicenter observational study	CRP, PCT	331	ICU	Respiratory	54.1
van Berkel (2020)^67^	The Netherlands	HIC	Not specified	Retrospective chart review	CRP, PCT	66	ICU	–	50
Vanhomwegen (2021)^68^	Belgium	HIC	03/03/2020–02/06/2020	Retrospective observational	PCT	66	ICU	Both	11
Vaughn (2021)^69^	USA	HIC	13/03/2020–18/06/2020	Retrospective observational	CRP, PCT	1,705	Both	Both	CO: 3.5
Williams (2021)^70^	UK	HIC	05/03/2020–15/04/2020	Retrospective observational	PCT	368	Non-ICU	–	–
Zhu (2023)^71^	China	UMIC	14/12/2022–30/01/2023	Retrospective observational	CRP	716	Both	Respiratory	73.9

–, not recorded; BC, blood culture; BAL, bronchoalveolar lavage; CO, community onset; CRP, C-reactive protein; HAI, hospital-associated infection; HIC, high-income country; HO, hospital-onset; ICU, intensive care unit; LMIC, low-middle-income country; PCT, procalcitonin; RC, respiratory cultures; UK, United Kingdom; UMIC, upper middle-income country; USA, United States of America; VAP, ventilator-associated pneumonia.

Most studies started and ended in 2020, during the first 2 waves of the pandemic. Several studies ended in 2021, 2 studies reported from 2022, and for 1 study, the study period was not specified. Several studies spanned multiple years. Although the majority were retrospective observational studies, there were 8 prospective studies and 1 randomized controlled trial (RCT).

The study population varied from a minimum of 49 to a maximum of 4,635 patients. One study specifically looked at the pediatric population, while the majority excluded patients <18 years old. Fifteen studies were specifically reported on patients admitted to an ICU. Ten studies investigated only respiratory co-infections, while most studies investigated both respiratory and non-respiratory co-infections.

The prevalence of microbiologically confirmed secondary bacterial infection varied from 1% to 60.6% with 5 studies not reporting secondary bacterial infections (Table 1).

Overall, for mild cases of COVID-19, most studies concluded positively for the use of biomarkers to rule out bacterial co-infections but should be interpreted with caution or in a multimodal approach with clinical assessment. However, most studies involving patients with severe COVID-19 concluded negatively on the use of PCT and bacterial co-infections; 2 studies^64,66^ described the use of CRP and/or PCT with an algorithm score, and 1 study^28^ investigated the kinetics of biomarkers for ICU-acquired infections.

### PCT results

There were 53 studies that assessed PCT levels and their relation with bacterial co-infections; 29 studies measured PCT with CRP levels, and 24 measured only PCT. Twenty studies reported negative predictive values (NPV), positive predictive values (PPV), and/or sensitivity and specificity for bacteria co-infections according to the chosen PCT cut-off values (Table 2).^7,19,20,23–25,31,32,36,40,44,51,54,56,57,60,61,67–69^

**Table 2.** Cut-off values, negative predictive values (NPV), positive predictive values (PPV), sensitivity, and specificity results for procalcitonin (PCT) in identifying bacterial co-infections in patients with coronavirus disease 2019

First author (year)	Cut-off values	NPV	PPV	Sensitivity	Specificity
Atallah (2022)^19^	<0.25 ng/mL	BSI: 95.3% bPNA: 95.6%	BSI: 31.3% bPNA: 18.6%	BSI: 82% (0.25 ng/ml) bPNA: 86% (0.5 ng/mL)	BSI: 70% (0.25 ng/ml) bPNA: 44% (0.5 ng/mL)
Azijli (2022)^20^	<0.10 ng/mL <0.25 ng/mL <0.5 ng/mL	0.25 μg/L: 100% (95% CI, 63.1–100)	0.25 μg/L: 11.0 (95% CI, 9.17–13.1)	0.25 μg/L: 100%	0.25 μg/L: 68.0% (95% CI, 61.1–74.4)
Campani (2023)^23^	Best cut-off value determined from ROC curve: ≥0.16 ng/mL	≥0.16: 63.1 (95% CI, 54.3–71.3) ≥0.25: 58.2 (95% CI, 50.1–66.0)	≥0.16: 82.2 (95% CI, 75.0–88.0) ≥0.25: 85.5 (95% CI, 77.5–91.0)	≥0.16: 71.0 (95% CI, 63.5–77.7) ≥0.25: 60.9 (95% CI, 53.2–68.3)	≥0.16: 76.4 (95% CI, 67.3–83.9) ≥0.25: 83.6 (95% CI, 75.4–90.0)
Carbonell (2022)^24^	0.50 ng/mL	<0.3 ng/mL: 91.1% (95% CI, 90.0–92.2)			
Ceccarelli (2023)^25^	0.5 ng/mL	63.8% (95% CI, 60.5–67.0)	37.4% (95%CI, 25.9–51.8)	19.2% (95% CI, 10.1–33.3)	81.6% (95% CI, 76.4–85.9)
Cowman (2022)^31^	>0.5 ng/mL ≤0.5 ng/mL	>0.25 ng/mL: 96% >0.5 ng/mL: 94%	>0.25 ng/mL: 13% >0.5 ng/mL: 13%	>0.25 ng/mL: 82% >0.5 ng/mL: 58%	>0.25 ng/mL: 47% >0.5 ng/mL: 61%
Dolci (2021)^32^	<0.25 ng/mL ≥1.0 ng/mL		Initial^a^: 58.8 (95% CI, 45.9–70.7) Peak^b^: 65.4 (95% CI, 49.0–80.7)	Initial^a^: 60.6 (95% CI, 42.1–77.1) Peak^b^: 51.5 (95% CI, 33.5–69.2)	Initial^a^: 72.0 (95% CI, 57.5–83.8) Peak^b^: 82.0 (95% CI, 68.6–91.4)
Galli (2023)^36^	Best cut-off value determined from ROC curve: ≥0.12 ng/mL	≥0.12: 97.5 (95% CI, 96.5–98.5) ≥0.25: 97.3 (95% CI, 96.6–98.0)	≥0.12: 3.5 (95% CI, 2.9–4.2) ≥0.25: 3.8 (95% CI, 3.0–4.7)	≥0.12: 81.2 (95% CI, 74.2–88.2) ≥0.25: 59.4 (95% CI, 50.7–68.1)	≥0.12: 24.9 (95% CI, 23.5–26.3) ≥0.25: 49.5 (95% CI, 47.9–51.0)
He (2021)^40^	<0.5* ≥0.5*			0.66	
Houghton (2021)^44^	<0.25 ng/mL 0.25–0.5 ng/mL ≥0.5 ng/mL	Baseline (0.5): 97.6% Baseline (0.25): 90.2% 48 h (0.5): 100% 48 h (0.25): 89.7%	Baseline (0.5): 24.7% Baseline (0.25): 39.4% 48 h (0.5): 33.3% 48 h (0.25): 50.5%	Baseline (0.5): 94.6% Baseline (0.25): 87.5% 48 h (0.5): 100% 48 h (0.25): 90.3%	Baseline (0.5): 42.8% Baseline (0.25): 46.3% 48 h (0.5): 43.9% 48 h (0.25): 48.6%
Malinverni (2022)^7^	<0.1 ng/mL 0.1–0.249 ng/mL 0.25–0.49 ng/mL >0.5 ng/mL	≥0.25 ng/mL: 76.7 (95% CI, 69.7–82.8) ≥0.5 ng/mL: 79.7% (95% CI, 73.8–84.8)	≥0.25 ng/mL: 31.1% (95% CI, 22.9–40.2) ≥0.5 ng/mL: 46.4% (95% CI, 34.3–58.8)	≥0.25 ng/mL: 48.1% (95% CI, 36.5–59.7) ≥0.5 ng/mL: 41.6% (95% CI, 30.4–53.4)	≥0.25 ng/mL: 61.7% (54.8–68.2) ≥0.5 ng/mL: 82.7% (77.0–87.5)
May (2021)^51^	0.25 ng/mL 0.5 ng/mL	Bacteriuria: 0.970, BSI: 0.988, bPNA: 0.995	Bacteriuria: 0.043, BSI: 0.027, bPNA: 0.015	Bacteriuria: 0.568, BSI: 0.681, bPNA: 0.708	Bacteriuria: 0.527, BSI: 0.528, bPNA: 0.526
Moreno-García (2022)^54^	≥0.2 ng/mL ≥0.5 ng/mL ≥1 ng/mL ≥2 ng/mL	≥0.2 ng/mL: 0.92 ≥0.5 ng/mL: 0.92 ≥1 ng/mL: 0.92 ≥2 ng/mL: 0.92	≥0.2 ng/mL: 0.12 ≥0.5 ng/mL: 0.14 ≥1 ng/mL: 0.21 ≥2 ng/mL: 0.34	≥0.2 ng/mL: 0.40 ≥0.5 ng/mL: 0.19 ≥1 ng/mL: 0.14 ≥2 ng/mL: 0.14	≥0.2 ng/mL: 0.71 ≥0.5 ng/mL: 0.89 ≥1 ng/mL: 0.95 ≥2 ng/mL: 0.97
Nazerian (2021)^56^	≥0.5 ng/mL	60% (49.4–67.9%)	57.2% (39.9–72.9%)	42.9% (24.5–62.8%)	71.9% (53.3–86.3%)
Ng (2022)^57^	≥0.11 ng/mL			48.6%	73.5%
Pink (2021)^60^	0.55 ng/mL	94%	69%	91%	81%
Relph (2022)^61^	0.25 ng/mL 0.5 ng/mL			0.25 ng/mL: 59.4% (95% CI, 52.8–65.6) 0.5 ng/mL: 44.2% (95% CI, 37.6–50.9)	0.25 ng/mL: 50.4% (95% CI, 46.9–53.8) 0.5 ng/mL: 65.4% (95% CI, 62.1–68.7)
van Berkel (2020)^67^	<0.5 ng/mL	0.25 ug/L: 81% 0.5 ug/L: 62%	0.25 ug/L: 65% 0.5 ug/L: 66%	0.25 ug/L: 90% 0.5 ug/L: 66%	0.25 ug/L: 46% 0.5 ug/L: 62%
Vanhomwegen (2021)^68^	≥ 0.5ng/mL			71%	43%
Vaughn (2021)^69^	0–0.1 ng/mL 0.1–0.25 ng/mL 0.25–0.5 ng/mL >0.5 ng/mL	>0.1 ng/mL: 98.3% (CO)	>0.5 ng/mL: 9.3% (CO)		

bPNA, bacterial pneumonia; BSI, bloodstream infection; CO, community onset.

a PCT cut-off >0.8 ng/mL.

b PCT cut-off >2.5 ng/mL.

* No units reported.

PCT cut-off values varied from 0.1 ng/ml up to 2 ng/ml; in 10 studies, multiple cut-off values were considered, where most studies considered 0.25 ng/mL as the lower cut-off value and 0.5 ng/mL as the upper cut-off value. Two studies established cut-off values by using receiver operating curves (ROC) to determine a sensitivity of 80%.^23,36^ In general, studies with lower cut-off values reported higher NPVs, and studies with higher cut-off values reported higher PPVs for bacterial co-infections. NPVs ranged from 58.2% to 100% using ≤0.25 ng/mL as the cut-off. PPVs ranged from 3.5% using 0.12 ng/mL to 85.5% using 0.25 ng/mL as cut-offs. There was a varied range of results for sensitivity and specificity for detecting bacterial co-infections across the board with no obvious trends (Table 2).

### CRP results

Thirty-four studies measured CRP levels in COVID-19 patients; 4 measured CRP levels only, while 29 measured both CRP and PCT. Reported cut-off values ranged from 65mg/L to 312.5 mg/L; 1 study did not report a cut-off value but did report a sensitivity result^40^, and 1 study provided cut-off values for the initial result and 1 for the peak result^32^. Two studies determined cut-off values from using ROC to determine a sensitivity of 80%.^23,36^

### Antimicrobial stewardship (AMS)

Of the 59 studies reviewed, 8 studies reported clear objectives around AMS in their design and were further analyzed (Table 3).^9,17,34,35,53,58,64,70^ Five were retrospective studies and conducted in HICs (UK, USA);^9,35,53,58,70^ there was 1 prospective study conducted in a UMIC (Thailand), a before-and-after observational study in a HIC (USA),^17^ and an RCT from a HIC (France)^34^. All studies used PCT as a biomarker, with a cut-off of 0.25 ng/mL, except in 2 studies where there were categories of cut-offs (<0.25 ng/mL, ≥0.25–<0.5ng/mL, ≥0.5ng/mL),^34,58^ and 1 study used 0.5 μg/L as the cut-off.^64^ Two studies only included patients admitted in ICU, 2 studies had both ICU and non-ICU patients, while the other 4 studies only included patients admitted in general wards.

**Table 3.** Antimicrobial stewardship and safety outcomes for studies reporting usage of procalcitonin (PCT)-guided prescription guidelines

Author (year)	When PCT measured/used? How?	AM stewardship outcomes	Safety outcomes
Anderson (2023)^17^	<0.25 ng/mL with active AM use	Less AM prescribed (*P*: 0.002) Reduced AM duration (*P*: 0.034)	No statistical difference in LoS, inpatient mortality, *Clostridioides difficile* infections, AM reinitiation, or AM prescribed at discharge
Calderon (2021)^9^	Measured within 72 h AM start	PCT group had lower AM exposure and consumption PCT group adjusted mean AM duration (adjusted ROM = 0.70; 95% CI, 0.6–0.9) and adjusted mean DDD (ROM = 0.70; 95% CI, 0.6–0.8) both 30% lower AM consumption in PCT group reduced over time	PCT group less likely to die within 30 d (adjusted PR: 0.6; 95% CI, 0.4–1.1) No differences in: LoS Admission to HDU/ICU 72 h after starting AM Readmission to hospital within 30 d
Fartoukh (2023)^34^	As soon as possible after randomization into the trial (following admission to ICU)	81% adherence to intervention protocol At D28, no difference in median number of AM free days (*P*: 0.89) No difference in incidence of administration of new AM for first clinical suspicion of bacterial superinfection at D28, considering the competing event of death (sHR: 1.22 [95% CI, 0.83– 1.79]; *P*: 0.31; n: 190)	No difference in LoS or mortality rate at D28 and D90 Intervention group 2.5% less infection with *C. difficile* or multidrug-resistant bacteria
Fratoni (2022)^35^	Measured prior to AM initiation	Use of PCT by prescribers was ubiquitous in the study population (99.9%) 60% AM discontinued within 24 h following <0.25 ng/mL PCT result Pharmacist intervened in 13 patient care despite <0.25 ng/mL PCT result = 9 patients AM discontinued	No difference in LoS, *C. difficile* infections, mortality rate, discharge disposition or 30-d readmission
Moore (2022)^53^	First PCT value >0.5 ng/mL	Overall acceptance rate of AMS was 74.2% o 86.8% acceptance for early discontinuation o 54.2% acceptance for late discontinuation	No difference in mortality or median LoS between early and late AM discontinuation High initial AM prescription rate
Peters (2020)^58^	PCT used in cases where bacterial co-infection could not be ruled out	72.5% of COVID-19 cases with PCT <0.25 μ/L either never had AM or had AM stopped within 48 h PCT reduced AM use by 44%	None reported
Sathitakorn (2022)^64^	PCT ordered on admission and on Day 3	Less inappropriate empirical AM initiation (58.3% vs 100%; *P* < 0.01) More AM discontinued at 72 h (13.3% vs 0%; *P* < 0.01) Significantly shorter total AM duration (2 d vs 7 d; *P* < .01)	Significant reduction in median LoS (10 d vs 12 d; *P* < 0.01) No significant difference for 30-d or infectious disease-related mortality between groups
Williams (2021)^70^	PCT collected within 48 h of positive COVID-19 sample If ≤0.25 ng/mL: withhold AM	Compliance with guidelines at 67% for negative PCT and 84% for positive PCT Significantly less DDDs prescribed to negative PCT group (median DDD: 3.0 vs 6.8; *P* < 0.001)	Significant reduction in mortality (28% vs 36%; *P*: 0.021) Non-significant reduction in LoS No cases of *C. difficile* in either group No difference in infective complications between groups No negative impacts on 28-d outcomes

AM, antimicrobial; AMS, antimicrobial stewardship; COVID-19, coronavirus disease 2019; DDD, defined daily dosage; LoS, length of stay; ROM, ratios of means.

Two of 8 studies directly compared measures of antimicrobial use in a group that incorporated PCT guidance versus a group without PCT measurements.^9,34^ All 4 studies that compared defined daily doses (DDD) of antimicrobial treatment observed a significant reduction in the PCT group versus the group without PCT guidance^9,17,58,70^. Six studies compared the length of antimicrobial treatment in groups with and without PCT guidance; 4 of these demonstrated a reduction when PCT was used, and 2 did not detect a difference between groups.^9,17,34,35,64,70^

Similarly, different secondary measures of safety were used in comparative studies. In 6 studies reporting on mortality rate, no adverse effect of PCT-guided AMS was detected,^17,34,35,64^ and a reduction was seen in 2 studies.^9,70^ Of the 7 studies that measured the length of stay (LoS),^9,17,34,35,53,64,70^ 2 observed a reduction in the PCT group,^64,70^ and no difference was detected in the remaining studies. Four studies measured *Clostridioides difficile* infections,^17,34,35,70^ and 1 observed a nonsignificant reduction in incidence with PCT-guided AMS.^34^

### LMIC studies

Only 3 studies were reported from LMICs.^21,49,55^ All studies were retrospective descriptive studies and were performed at a teaching/university hospital.

The study from Nepal by Basnet *et al* ^21^ describes a cross-sectional study investigating the prevalence of uropathogenic *Escherichia coli* among COVID-19 patients admitted to tertiary care. Of the 49 COVID-19 patients with symptoms of a urinary tract infection, 3 had uropathogenic *E. coli* (6.1%) detected. The mean PCT levels were higher for co-infected patients than not (6.13 ng/mL vs 0.95 ng/mL, respectively). It is unknown if the patients were in ICU or had severe COVID-19, which may have affected the PCT levels.

The study from Pakistan by Nasir *et al* ^55^ describes a retrospective case-control study of 50 COVID-19 patients with a confirmed bacterial infection matched to COVID-19 patients without bacterial co-infection. Patients were from both the ICU and normal medical wards. Almost ¾ of co-infections were hospital-acquired (72%), with the majority being hospital-acquired pneumonia. Compared to patients without an infection, there was no significant difference in CRP or PCT on logistic regression analysis. Although there were no significant results for these host biomarkers, the report highlights the need for AMS, as 64% (32/50) of patients without a confirmed infection received antibiotics.

The study from India by Lukose *et al* ^49^ evaluated the patterns and predictors of empirical antibiotic therapy in patients admitted for moderate and severe COVID-19. Elevated PCT [OR: 3.91 (95% CI, 1.66–9.16) (*P* = 0.001)] levels were identified as predictors for initiating empirical anti-bacterial therapy, but no specific cut-off values were identified.

## Discussion

This scoping review provides an overview of where and how biomarkers were used throughout the first waves of the COVID-19 pandemic to assist with AMS efforts. Procalcitonin has the potential to help in diagnosing bacterial co-infections in patients with COVID-19; however, the predictive values (NPV/PPV) are inadequate for the tests to be used in isolation and results to be interpreted together with other clinical information.

Most identified studies considered PCT at 0.25 ng/mL as the cut-off value for withholding antibiotic prescriptions, with some studies using 0.5 ng/mL as a higher cut-off value—often studies within the ICU. This is similar to what was reported in a similar review from earlier in the pandemic; Omer *et al* ^8^ reported that half of the studies used 0.5/0.55ng/mL and another third used 0.2/0.25 ng/mL. However, in a meta-analysis of 8 studies using a PCT cut-off of 0.5 ng/mL to distinguish between bacterial and viral CAP prior to the COVID-19 pandemic, Kamat *et al* ^72^ concluded that the sensitivity and specificity estimates are too low to confidently use this PCT cut-off in decision-making processes.^73^

In this review, there were a limited number of studies evaluating CRP, with a wide range of cut-off values from the 5 studies using CRP alone. There was no consensus on cut-off values from the studies reporting CRP, and furthermore, there are several confounding issues with COVID-19, inflammation and CRP.

Interestingly, several studies propose the use of CRP and PCT in combination with other inflammatory markers and clinical scores.^40,51,54^ When using a clinical pulmonary infection score with a PCT cut-off of 0.5 ug/L in severely ill COVID-19 patients, Sathitakorn *et al* ^64^ reported those with a negative score were less likely to have inappropriate antibiotics used, less likely to have inappropriate empirical antibiotic initiated, and more likely to have antibiotics discontinued at 72 hours. In their retrospective analyses, both Gianella and Tanzarella *et al* used the clinical findings to develop a predicative model for bacterial pneumonia diagnosis: Gianella *et al* in all COVID-19 patients and Tanzarella *et al* in severe COVID-19 patients.^38,66^ Both studies also include PCT (≥0.2 ng/mL) and WBC in their scores. Therefore algorithms with several biomarkers and clinical scores may overcome the limitations of individual biomarker interpretation.

Our search only identified 3 retrospective studies conducted in LMICs; all were reported from Asia, specifically in tertiary hospitals/teaching hospitals with better access to diagnostic facilities. Due to differing study populations, small study population sizes in 2 of the 3 studies, no clearly defined cut-off values, different conclusions, and no strong recommendations, there can be no overarching inferences made for the use of biomarkers for COVID-19 patients in LMICs. However, a recent review by Lamrous *et al* in non-COVID-19 LMIC contexts suggests that PCT is likely to be as reliable a clinical tool in LMICs as in HICs, particularly in respiratory tract infections, sepsis, and HIV/TB.^2^ However, more studies are needed to reach a consensus regarding laboratory standards and cut-off values.

We identified a lack of representation from other geographical areas such as Africa and Latin America, where the different epidemiology of potential co-infections (malaria, dengue, etc.) on biomarkers behaviors in COVID-19 has not been reported. Although there are potential host and pathogen response differences for PCT and CRP in the presence of LMIC geographical specific endemic infections, this is unlikely to dramatically influence their dynamics in the context of COVID-19.

Overall, there were few studies that documented the direct integration of biomarkers into AMS programs and none from LMICs. From the 5 studies that specifically reported AMS outcomes in this review, there was an overall decrease in antibiotic consumption with no impact on the measures of safety reported for mild COVID-19 cases (Table 3). Thirteen of the 18 studies in the Omer *et al* review indicated positively the use of PCT for ruling out superimposed bacterial infection(s) and/or as an AMS tool, while in the Wolfisberg *et al* review found that for COVID-19 specifically, most studies reported reduced antibiotic use with no negative impacts on outcomes.^4,8^ The MultiCoV RCT used a respiratory multiplex Polymerase Chain Reaction (PCR) panel and PCT algorithm to reduce antibiotic exposure in patients with severe confirmed COVID-19 pneumonia and reported no significant differences in serious adverse events or mortality rate between the PCR/PCT algorithm and conventional strategies.^34^ Ultimately, the scarcity of articles in this review highlights the need for more trials and implementation research, particularly in the context of COVID-19 and low-resourced settings.

However, the best AMS algorithms are only as good as the compliance rate, with consistent education key.^3,74^ In an evaluation of an AMS program with PCT guidelines in the UK, Williams *et al* ^70^ found that one-third of patients in the negative PCT (≤0.25 ng/ml) group were on antibiotics 48 h after a COVID-19 diagnosis, compared to 84% of patients with a positive PCT (>0.25 ng/mL) result. In a qualitative study investigating hospital physicians’ experiences with using PCT in an AMS algorithm in Norway (prior to COVID-19), physicians reported a knowledge gap in usage, expressing uncertainty of usage and interpretation, with some clinicians describing experiences where PCT failed to indicate a bacterial infection and thereby increased their lack of confidence in PCT as an indicator.^74^ The transition from the evidence of biomarkers to the practice of using them within AMS programs needs to be explored further with implementation research.

There are limitations to this review considering the objectives of this study. First, we did not consider the interaction of immune modulators with biomarkers in COVID-19 patients. The use of dexamethasone, tocilizumab, or baricitinib may confound the interpretation of host inflammatory markers and thereby limit the diagnostic performance of biomarkers. Studies have concluded that in critically ill COVID-19 patients, CRP and PCT have shown rebound increases upon cessation of immunomodulator treatment, and as such, clinicians should assess basic clinical infection signs and cultures for diagnosis of secondary bacterial infections.^3,75^

Most studies were retrospective single-center studies, conducted during the first wave of COVID-19; most studies in this review were performed in 2020, particularly early/mid-2020, and before mass vaccination campaigns. Subsequent variants of SARS-CoV-2 and vaccination coverage have resulted in infections with different transmissibility, epidemiology, hospitalization, and mortality rates.^76^

Most studies in this review were conducted in HICs with better laboratory capacity to aid the diagnosis of bacterial co-infection. Only 3 studies were performed in LMICs, and there was a lack of representation from Africa and Latin America, where there are different endemic diseases that may play a role in the dynamics of biomarkers.^2^

Finally, although there was a larger proportion of studies that used 0.25 ng/mL as a PCT cut-off, there needs to be a clear consensus on biomarker cut-offs and what that cut-off determines—whether that be the prescription, the de-escalation, or the withdrawal of antimicrobial agents. Larger, multicenter studies need to be performed to provide clear evidence for this decision; the current BATCH and PEACH trials will hopefully add to the necessary evidence to make these decisions.^77,78^

## Conclusion

In the context of non-ICU hospitalized COVID-19 cases in HMICs, a PCT cut-off value below 0.25 mg/L can be a useful tool to rule out bacterial co-infection, but the wide range of NPVs reported in this review suggests that PCT should be interpreted in the context of other clinical findings. However, from this review, there is too little data to be conclusive about the use of CRP in the same manner. AMS programs in the right clinical context can incorporate a PCT value of <0.25 mg/L as a cut-off for the administration of antibiotics in mild COVID-19 patients without concerns for adverse outcomes. Although non-COVID-19-specific evidence suggests that the use of PCT in this manner should be safe in LMICs, local scientific institutions, international research partnerships, and humanitarian organizations can play an essential role to pilot the use of PCT as an antibiotic stewardship tool in the COVID-19 context.
